# Canalization of neural dynamics by δ-protocadherins in the developing zebrafish optic tectum

**DOI:** 10.1371/journal.pgen.1012171

**Published:** 2026-06-01

**Authors:** Sayantanee Biswas, Michelle R. Emond, Grace S. Philip, James D. Jontes

**Affiliations:** Department of Biological Chemistry and Pharmacology, Ohio State University Wexner College of Medicine, Columbus, Ohio, United States of America; Fred Hutchinson Cancer Research Center, UNITED STATES OF AMERICA

## Abstract

Brain dynamics are constrained by the underlying topology of neuronal networks. How genes collaborate to organize these neural networks during development remains an enduring mystery. In humans, large numbers of genes have been implicated in neurodevelopmental disorders that are characterized by variable and overlapping phenotypes. The complexity of the brain and the heterogeneity of the disorders makes understanding the relationships between genes, development and neural function challenging. Beginning in the 1940s, Waddington suggested the concept of canalization to describe the role of genes as buffering developmental trajectories against genetic and environmental variation, leading to precise outcomes. Here, we show that members of the δ-protocadherin family of homophilic cell adhesion molecules, Protocadherin-19 and Protocadherin-17, contribute to developmental canalization of neural dynamics in the visual system of larval zebrafish. We provided oriented visual stimuli to zebrafish larvae and performed *in vivo* 2-photon calcium imaging in the optic tectum. The latent dynamics resulting from the population activity were remarkably conserved among different wild type larvae, allowing quantitative comparisons within and among genotypes. In both Protocadherin-19 and Protocadherin-17 mutants, the latent dynamics diverged stochastically from wild type, suggesting that the loss of these adhesion molecules leads to stochastic phenotypic variability and introduced disruptions of circuit organization that varied among individual mutants. These results are consistent with the developmental canalization of a vertebrate neural circuit, and suggest a framework for understanding the observed variability in complex brain disorders.

## Author summary

Mutations in a wide array of genes can contribute neurodevelopmental disorders, such as autism spectrum disorders or schizophrenia. The effects of mutations can be weak and variable, making it challenging to study their roles in these disorders. Here we image neural activity in the brain of living zebrafish larvae in response to visual stimulation. We find that the direction of motion is encoded in the coordinated activity of neurons, and that the response is remarkably similar among wild type individuals. The stereotyped responses allow us to use population neural activity to quantitatively compare zebrafish harboring mutations in different genes. We show that mutations in genes encoding the cell adhesion molecules, Pcdh19 or Pcdh17, cause defects in visual processing. In both cases, the defects vary stochastically among the mutants, suggesting that these cell adhesion molecules limit variability in the assembly of the underlying neural networks. Our results provide an approach for quantifying phenotypic variability in a developing neural circuit, as well as the means to explore the combined effects of multiple mutations.

## Introduction

The organization of biological neural networks is determined by the coordinated action of conserved genetic and experience-dependent mechanisms that have been refined through evolution.This assembly process is robust to genetic and environmental variation, as well as natural biological heterogeneity, allowing the brains of distinct individuals to perform the same tasks, even though they are not identical at the level of neurons and synapses. To fully understand neural development, it is necessary to account for the resilience of phenotype to genetic and environmental variation. Addressing this issue, Waddington introduced the concept of canalization [4,5]; through natural selection, developmental processes have evolved to buffer against small environmental, genetic or stochastic perturbations to bring about a most probable outcome. He imagined troughs within an epigenetic landscape, shaped by the collective action of genes, that would guide developmental trajectories. However, there are limits to robustness in brain development, as mutations in some genes can lead to neurodevelopmental disease, which could be viewed as instances of decanalization [6,7].

Many genes have been linked to neurodevelopmental diseases, such as schizophrenia and autism spectrum disorders. In some cases, rare, deleterious mutations in genes are causal for neurodevelopmental disease, such as *MECP2* in Rett Syndrome and *FMR1* in Fragile-X syndrome [8–10]. However, in most cases, mutations exhibit low penetrance and only confer an increased risk. The most common explanation is that these diseases are polygenic; the impact of individual risk alleles may be negligible on their own, but the cumulative effect of multiple risk alleles can reach a threshold for manifesting a disorder [2,11]. Moreover, individual risk genes exhibit variability in penetrance and expressivity [3] and can be associated with multiple disorders [1,2,12]. Thus, mutations in a large number of clinically relevant genes may exhibit little discernible phenotype in humans or animal models. The complexity of neurodevelopmental disease is further exacerbated by phenotypic heterogeneity [13]. Collectively, these sources of variation mask the relationships between gene function, circuit organization and neural activity. To study the effects of risk genes whose phenotype may be subtle, it is necessary to have sensitive, quantitative assays of neural function that are capable of distinguishing aberrant phenotype from natural biological variation.

Protocadherin-19 is a member of the δ-protocadherin family of homophilic cell adhesion molecules [14–16]. In humans, mutations in *PCDH19* are linked to schizophrenia and autism [17,18], and cause a female-limited developmental epileptic encephalopathy (*PCDH19*-FE) [19,20]. The related gene *PCDH17* has been linked to major mood disorders [21]. Prior work has shown that loss of *pcdh19* disrupts the development of network topology [22] or excitability in zebrafish and network synchronization or excitability in rodents [23,24]. We have also previously shown that zebrafish larvae lacking *pcdh19* exhibit defects in visually-guided behaviors [25]. To investigate how mutations in *pcdh19* or the related *pcdh17* affect the development of neural dynamics, we provided a well-defined pattern of visual stimulation while using *in vivo* 2-photon calcium imaging to record neural activity in the optic tectum of the larval zebrafish. We show that the neuronal population responses to oriented sinusoidal gratings define neural dynamics that are strongly stereotyped among wild type larvae. In mutants, these dynamics are altered, reflecting stochastic deviations in the underlying circuit organization; each individual mutant responds consistently, but in a way that varies stochastically within the population. Our results are consistent with a role for protocadherins in canalization of nervous system development, contributing to robustness of visual processing. This suggests that the effect of disease-linked mutations in an individual does not have a specific consequence or give rise to a particular circuit defect, but allow stochastic deviations in brain connectivity. These random variations could represent decanalization of neural development, and this phenotypic variation could provide the basis for the variability and polygenicity observed in neural disorders.

## Results

**Fig 1:**
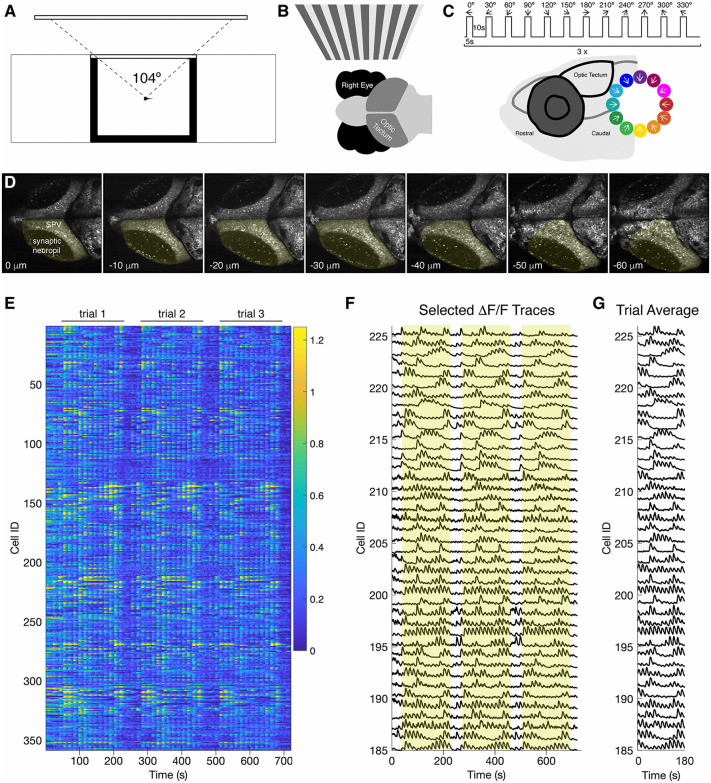
Calcium-imaging of neural responses to oriented sinusoidal gratings. A. Schematic of the experimental setup. A 6dpf larva is embedded in agarose in an imaging chamber, allowing 104° view of a projection screen. B. The visual stimuli are presented to the right eye and the neural responses are visualized in the contralateral (left) hemi-tectum. C. The stimulation protocol is a series of 5s on 10s off of a moving sinusoidal grating, 12 directions spaced at 30° intervals to encompass the full 360°. This is repeated three times. Below is a lateral view of the head of a zebrafish larva. The inset shows the directions of motion of sinusoidal gratings with reference to the rostro-caudal axis of the fish. The direction color-code is used throughout the paper. D. Image stacks consisting of 7 optical sections spaced at 10 μm were collected at 1s intervals. Shown are average intensity images of individual planes from a timelapse sequence collected in a 6 dpf transgenic larva, Tg(elav3l:HistoneH2B-GCaMP8s). The visually-responsive hemi-tectum is outlined in yellow. E. Example data showing all the fluorescence traces from visually-responsive neurons collected from one larva. F. ∆F/F traces selected from those shown in E. Each 180 s trial is highlighted with yellow boxes. The full timecourse is 735 s. G. Responses were averaged across the three trials (180 s). Shown are the trial averages from the traces in F.

The zebrafish optic tectum is specialized for multisensory integration and sensorimotor transformation, and is important for behaviors such as prey tracking and predator avoidance [26,27]. The cell bodies of tectal neurons are organized in the *stratum periventriculare* (SPV) and project their processes to a laminated synaptic neuropil [28–33]. Retinal ganglion cell axons from the contralateral retina arborize in the superficial *stratum opticum* to provide inputs to the tectum [34–37]. Moving stimuli, such as lines, spots or gratings, elicit robust responses in the tectal neurons [33,38,39]. To record neural activity in the optic tectum, we generated a transgenic line that *pan*-neuronally expresses a nuclear localized jGCaMP8s [40] (Histone H2B-GCaMP8s). These transgenic larvae were mounted in a custom imaging chamber (Fig 1A), and we evoked visual responses by projecting a sequence of drifting sinusoidal gratings (12 directions rotated at 30° intervals) onto an adjacent translucent screen (Fig 1B and 1C). Two-photon image stacks spanning the optic tectum (7 optical sections at 10 μm spacing) were collected at 1 Hz (Fig 1D), allowing us to sample neural population activity (S1 Video). We used constrained non-negative matrix factorization (CNMF) with CaImAn [41] to automatically segment images and extract fluorescence traces from visually-responsive neurons (Fig 1E and 1F). For further analysis, the neural responses were averaged across three trials (Fig 1G). Overall, we obtained 2506 visually responsive neurons (n = 9 larvae).

**Fig 2:**
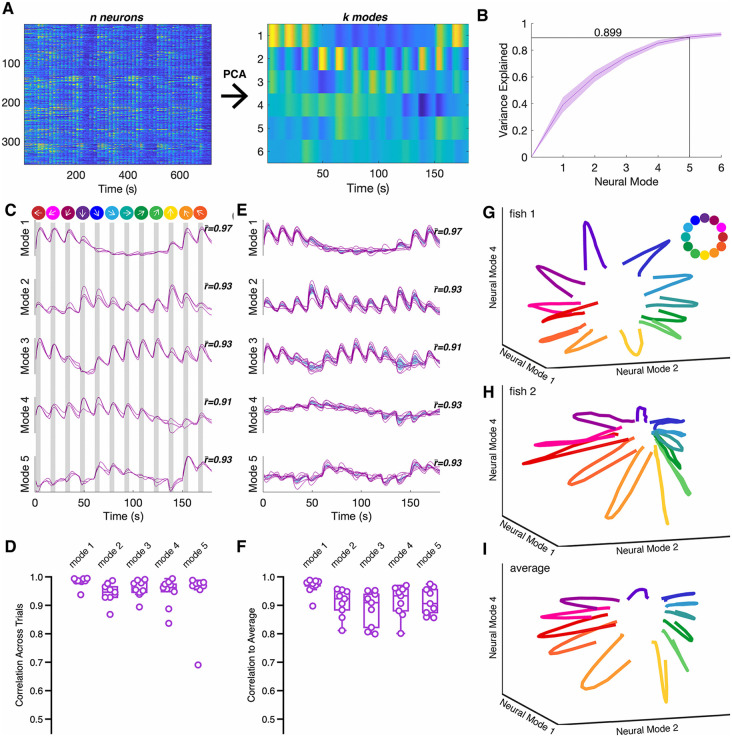
Latent dynamics in the optic tecta of zebrafish larvae in response to visual stimulation. A. Illustration of our approach to data analysis. Datasets such as the one shown on the left consisted of fluorescence traces from n neurons. These were trial averaged and the dimensionality of the trial averaged data was reduced using PCA. The new k dimensions are referred to as neural modes. B. The first 5 neural modes explain ~90% of the variance in the data. The shaded area represents the standard deviation, n = 9). C. Shown are the latent dynamics of an individual wild type larva for the first 5 neural modes. The latent dynamics were calculated independently for each of the three trials. The mean correlation of the dynamics among the individual trials to the trial average is shown as $\overset{―}{\text{r}}$. The presentation of each stimulus is shown as a gray bar, and the direction of each stimulus is shown at the top. D. Graph summarizing the mean correlations across trials along each mode for the wild type larvae (n = 9). E. Latent dynamics determined from trial averaged data. Traces (purple) are the dynamics from individual larvae and the shaded area (blue) is the variance for the wild type group. The value, $\overset{―}{\text{r}}$, represents the mean correlation of each individual neural mode to the mean dynamics averaged across the group (see f). F. Summary of the correlation between individual larvae and the group average along neural modes (n = 9). G,H. Three-dimensional representation of latent dynamics for two, individual larvae. Each color represents a direction of motion for the visual stimulus. I. A three-dimensional representation of latent dynamics averaged across the wild type group.

One emerging view of neural computation is that sensory processing and motor control are encoded by neuronal population dynamics, rather than the activities of individual neurons [42,43]. These latent dynamics consist of a small number of covariation patterns, or neural modes, which describe a neural trajectory within a low-dimensional subspace [44–46]. To explore the structure of tectal population dynamics in response to visual stimulation, we performed linear dimensionality reduction with principal component analysis (PCA) (Fig 2A). For n neurons, PCA reduces the data from an n-dimensional space to a more compact k-dimensional subspace. We found that the first five neural modes captured 89.9 ± 1.8% (n = 9 fish; mean ± sem.) of the variance in our wild type data (Fig 2B), and our subsequent analyses are confined to these five neural modes. Each neural mode represents a significant pattern of neuronal covariation that is activated to varying extent during the presentation of visual stimuli, and projection of the original neural data onto these axes describes the latent dynamics evoked by visual stimulation (Fig 2C). To determine how consistent the neural responses are in individual larvae, the latent dynamics were obtained for individual trials, and the Pearson’s correlation (r) between individual trials was calculated along each mode (Fig 2C and 2D). The mean trial-to-trial correlation was greater than 0.9 across all 5 neural modes (Fig 2D). Recent evidence suggests that the similarity of neural architecture among individuals can give rise to similar neural dynamics for a given stimulus or motor task [47]. In mammals, neural dynamics are estimated from a small subsample of neurons, which varies from individual to individual. In such cases, the neural dynamics from different animals need to be aligned using a procedure such as canonical correlation analysis [47,48]. Here, we image a relatively large proportion of visually-responsive neurons and sample among the same neuronal types across different fish, obviating the need for alignment (S1 Fig). For each wild type individual, we determined the neural dynamics from trial averaged responses (Fig 2E). To quantify the similarity of the population dynamics among the pool of wild types, we calculated Pearson’s correlation between an individual and the averaged dynamics computed from the other individuals in that group. (Fig 2E and 2F). The mean correlation along each neural mode was nearly as high as for trial-to-trial correlations (Fig 2C and 2D), indicating that the similarity of dynamics among individuals is comparable to the similarity of successive trials in the same individual. Thus, the latent dynamics elicited by visual stimulation is stereotyped across different zebrafish larvae, facilitating comparisons within and among experimental groups. When plotted in three dimensions, the latent dynamics from individual larvae described a rosette shaped trajectory, with each leaf representing the population response to a direction of grating motion (Fig 2G and 2H). The averaged dynamics yielded a smooth trajectory in three dimensions (Fig 2I). Thus, the population response to our simple stimulus presentation encodes the direction of motion within a reduced neural space that is preserved across zebrafish larvae.

**Fig 3:**
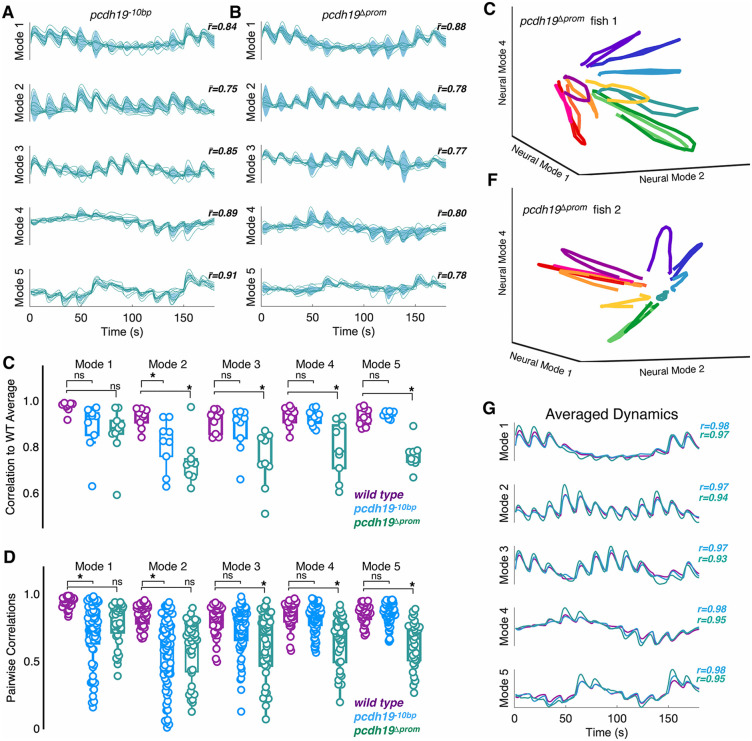
Mutations in *pcdh19* lead to altered neural dynamics. A,B. Shown are latent dynamics determined from trial averaged neural data collected in homozygous *pcdh19*^-10bp^ (A) and *pcdh1*9^Δprom^ (B) mutant larvae. Individual traces are in teal and the shaded area in blue indicates the group variance. The value, $\overset{―}{\text{r}}$, represents the mean correlation of each individual neural mode to the group averaged neural mode. We obtained a total of 3589 visually responsive neurons from *pcdh19*^-10bp^ larvae, and 2172 neurons from *pcdh19*^Δprom^ larvae. C. Boxplot shows the correlation of individual trial-averaged neural modes to the wild type average (wild type, n = 9; *pcdh19*^-10bp^, n = 12; *pcdh19*^Δprom^, n = 9; *p < 0.05, Dunnett’s test). D. Pairwise within-group correlations of neural modes for each genotype (wild type,n = 36; *pcdh19*^-10bp^, n = 66; *pcdh19*^Δprom^, n = 36; *p < 0.05, Dunnett’s test). E,F. Three-dimensional representations of latent dynamics of individual *pcdh19*^Δprom^ mutant larvae. The view is the same as in Fig 2G-2I. G. Neural modes were averaged within each genotype (wild type/purple, *pcdh19*^-10bp^/blue, *pcdh19*^Δprom^/teal. The value, r, represents the correlation of each mutant average with the wild type average (*pcdh19*^-10bp^/blue, *pcdh19*^Δprom^/teal).

To investigate the involvement of *pcdh19* in the assembly of visual circuitry, we used two, distinct *pcdh19* mutant alleles. We previously introduced an indel lesion near the 5’ end of exon1 shortly after the sequence encoding the signal peptide [25,49], resulting in a complete lack of functional Pcdh19 (*pcdh19*^*-10 bp*^). In addition, we have generated a new “promoterless” *pcdh19* allele, which lacks ~4kb of genomic sequence, spanning the basal promoter, 5’UTR and the ATG start codon in exon 1 (*pcdh19*^*Δprom*^)(S2A and S2B Fig). While both alleles completely eliminate the production of Pcdh19 protein (S2C Fig), evidence suggests that indel mutations, like *pcdh19*^*-10 bp*^, can elicit compensatory mechanisms arising from nonsense-mediated RNA decay [50,51]. Both of these mutant lines were crossed into our *Tg(elavl3:H2B-GCaMP8s)* line. As above, we performed calcium imaging on homozygous mutants during visual stimulation (S2 Video and S3 Video files) and computed the neural dynamics for each mutant from the trial averages of visually responsive neurons (Fig 3A and 3B; *pcdh19*^*Δprom*^*: 2178 neurons, n = 9 fish; pcdh19*^*-10 bp*^*: 2940 neurons, n = 12 fish*). To determine whether the mutant groups differed from wild type in their visual responses, we correlated the trial-averaged dynamics from each individual mutant with the wild type group average along each neural mode (Fig 3C). The response to drifting sinusoidal gratings diverged from wild type for both *pcdh19*^*-10 bp*^ and *pcdh19*^*Δprom*^ mutants, as the trajectories of individual mutant fish were less correlated to the wild type average than were individual wild type fish (Fig 3C). To determine whether individual mutants described similar or distinct trajectories, we calculated pairwise correlations between larvae within an experimental group (Fig 3D). If mutants converged on a distinct, but stereotyped, circuit organization, then their intra-group correlation should be similar to the intra-group correlations of the wild type group. Alternatively, if the divergence of the mutants from wild type is due to stochastic variations, then the intra-group correlations should be lower and exhibit an increased variance. Consistent with stochastic variation, the *pcdh19* mutants exhibit lower intra-group correlations than wild type larvae (Fig 3D). When visualized in three dimensions, the trajectories from individual *pcdh19*^*Δprom*^ mutants differ dramatically from the stereotyped paths of wild type larvae (Fig 2G and 2H), and diverge from each other (Fig 3E and 3F). However, despite the variability of the individual mutants, averaging within a genotype suppresses this variation and gives rise to averaged dynamics that are strongly correlated with wild type (Fig 3G). This suggests that the effect of *pcdh19* loss on visual processing is best understood as the result of stochastic deviations in development, as the effects on circuit function can be suppressed by averaging across individuals (Fig 3G).

**Fig 4:**
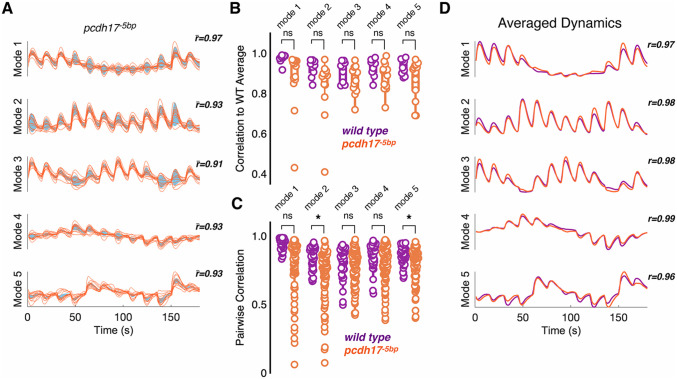
Mutations in *pcdh17* lead to altered neural dynamics. A. Latent dynamics computed from trial averaged neural data collected in homozygous *pcdh17*^-5bp^ mutant larvae. Individual traces are in orange and the shaded area in blue indicates the group variance. The value, $\overset{―}{\text{r}}$, represents the mean correlation of each individual neural mode to the group average of that neural mode. We obtained a total of 3561 visually-responsive neurons. B. The correlation of individual trial-averaged neural modes to the wild type average (wild type, n = 9; *pcdh17*^-5bp^, n = 13). C. Pairwise within-group correlations of neural modes for wild type (purple) and pcdh17 mutants (orange). (wild type, n = 36; *pcdh17*^-5bp^, n = 78; *p,0.05, Dunnett’s test). D. Neural modes were averaged for *pcdh17*^-5bp^ mutants (orange) and the average was compared to the wild type average (purple). The value, $\overset{―}{\text{r}}$, represents the correlation of each mutant average with the wild type average.

**Fig 5:**
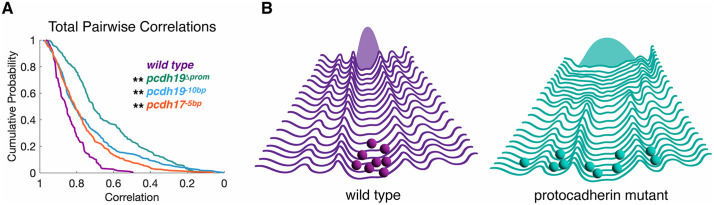
Mutations in pcdh19 or pcdh17 lead to increased phenotypic variability. A. Cumulative distribution of all pairwise within group 𝐫 values for wild type (purple), *pcdh19*^-10bp^ (blue) *pcdh19*^Δprom^ (teal), and *pcdh17*^-5bp^ (orange) mutants. (**p < 0.001, Dunn’s test). B. Hypothesized roles of Pcdh19 and Pcdh17 in canalyzing the assembly of visual circuits in the zebrafish optic tectum. In the wild type scenario (left/purple), development is canalized and the outcomes (circuit response to visual stimulation) is restricted, showing a small distribution in outcomes (shaded Gaussian). In the case of protocadherin mutants (right/teal), the developmental landscape is flattened, allowing development to stochastically explore a variety of alternative paths, leading to an increased variation among outcomes (broad shaded distribution).

Pcdh19 is a member of a small family of related δ2-pcdhs, which also includes Pcdh17. Both *pcdh19* and *pcdh17* are broadly expressed in the optic tectum, including both excitatory and inhibitory neurons (S3 Fig). To determine if mutations in other δ2-pcdhs similarly affect visual responses in the optic tectum, we recorded the response to visual stimulation in mutants lacking *pcdh17* (*pcdh17*^*-5 bp*^; *S4* *Video*) [49]. As above, we used the trial averaged neuronal responses to compute the latent dynamics along the first five neural modes (Fig 4A; *pcdh17*^*-5 bp*^*: 3523 neurons, n = 13 fish*). Similar to the *pcdh19* mutants, the latent dynamics exhibited by *pcdh17*^*-5 bp*^ mutants were less correlated with the wild type average (Fig 4A), although the differences were modest and not statistically significant (Fig 4B). To measure the intra-group variation exhibited by the *pcdh17*^*-5 bp*^ mutants, we determined pairwise correlations along each neural mode (Fig 4C). Although there was a uniform decrease in pairwise correlation among the *pcdh17*^*-5 bp*^ mutants, this was only significant for neural modes 2 and 5 (Fig 4C). As was observed for the *pcdh19* mutants, this intra-group noise was eliminated by averaging, as the *pcdh17*^*-5 bp*^ group average exhibited high correlation with the wild type average (Fig 4D). As each mutant genotype showed a reduced intra-group correlation, it is possible that this increased variation is due to unreliable or noisy neuronal responses. To determine whether variability in the tectal responses to visual stimulation was responsible for the variable phenotype, we determined the between-trial correlations for each neural mode within each experimental group (S4 Fig). We found no significant difference in the between-trial consistency of the population dynamics in the *pcdh17*^*-5 bp*^ and *pcdh19*^*-10 bp*^ mutant groups, compared to wild type (S4 Fig). There were statistically significant differences in neural modes 1 and 3 for the *pcdh19*^*Δprom*^ mutant, but these do not account for the observed phenotype; neural mode 1 is not significantly affected in the trial averages and neural modes 4 and 5 are affected in the trial averages, but don’t exhibit increased variability across trials. This suggests that the changes in the trial averaged data do not arise from increased trial-to-trial variability. To further quantify the within-group variance, we generated histograms of all pairwise correlation coefficients for all five neural modes within each experimental group (Fig 5A). While the distribution of correlation coefficients for the wild type group is narrow and concentrated at higher correlations, the mutants all exhibit significantly broader distributions (Fig 5A). Thus, loss of either *pcdh19* or *pcdh17* alters the neural population response to visual stimulation by introducing stochastic perturbations into circuit assembly. Our data are consistent with the idea that δ2-pcdhs contribute to canalization of the developing optic tectum (Fig 5B).

A common approach to investigating visual processing is to determine the tuning properties of individual neurons. To determine the effects of δ2-pcdh loss on neuronal properties, we determined an orientation selectivity index (OSI) and direction selectivity index (DSI), then compared the distributions of OSI (S5 Fig) and DSI (S6 Fig) for wild type and mutant larvae. Compared to wild type (S5A Fig), both the *pcdh19*^*Δprom*^ (S5B Fig) and *pcdh19*^*-10 bp*^ (S5C Fig) larvae exhibited a right shift toward increased OSI. In contrast, the *pcdh17*^*-5 bp*^ mutants exhibited decreased orientation selectivity (S5D Fig). In contrast, in each of the mutants, the DSI distribution shifted to the left, indicating a loss of direction selectivity (S6 Fig). For the direction selective neurons, we determined the preferred direction as a vector average of all the responses and generated polar histograms for wild type and mutant larvae (S7 Fig). Prior work has shown that direction selective neurons in the tectum largely respond to the four cardinal directions with the largest proportion of cells tuned to caudal-to-rostral motion [39,52–54]. While the rostral-to-caudal response was preserved in the mutants, each of the three mutants exhibited changes in the angular distribution of tuning preference compared to wild type.

In addition to homozygous mutants, we also analyzed the visual responses of heterozygotes (S8 Fig). Neural dynamics in the heterozygotes were similar to each of the respective mutants (S8A-S8C Fig; *pcdh19*^*Δprom*^*: 4621 neurons, n = 20 fish; pcdh19*^*-10 bp*^*: 2925 neurons, n = 14 fish; pcdh17*^*-5 bp*^*: 2838 neurons, n = 11 fish*). Both the correlation of individual neural modes to the wild type average (S8D Fig) and the pairwise, within-group correlations (S8E Fig) were lower than for wild type and the reduction was comparable in magnitude to what we observed in homozygous mutants. As was done for the homozygous mutants (Fig 5A), we generated histograms of all pairwise correlation coefficients within each experimental group, showing that the heterozygotes each exhibit significantly broader distributions, relative to wild type(S8F Fig).

## Discussion

Neural development is robust in that it can tolerate a significant amount of genetic and environmental variability yet still assemble a brain that interacts with the outside world in an appropriate manner. However, this robustness has limits, as many genes contribute increased risk for neurodevelopmental disease; as the impact of these mutations can be small and variable, it suggests that their effects on developmental trajectories are similarly variable. At the same time, experimental observations in animal models are subject to tremendous variation. For example, the numbers and distribution of neuronal types can vary among imaging experiments and neuronal responses are variable and noisy. Particularly in the study of risk genes with low penetrance and expressivity, it is necessary to disentangle the natural biological variation and experimental variation from the phenotypic variability arising from deleterious mutations.

Neural dynamics are constrained by the underlying patterns of neuronal connectivity [46], and it was recently shown that these low-dimensional dynamics are preserved across individuals [47]. Here, we show that the presentation of defined visual stimuli elicits neural population dynamics that can be described by a small number of neural modes. Each mode represents a pattern of covariation present within the neural population. The activation along each mode can roughly be ascribed to some stimulus features. For example, Mode 1 is most strongly activated by caudal-to-rostral directional motion. In contrast, Modes 2 and 3 appear to represent orientation selectivity for vertical and horizontal motion, respectively. These responses are remarkably similar across individual wild type larvae, as the variation in neural dynamics among distinct larvae is comparable to the variation among trials within the same animal. Thus, the complex neural responses of hundreds of neurons in different animals can be reduced to a common set of activity patterns that can be averaged and quantitatively compared within and between genotypes. The low variation among wild type individual larvae indicates that, within a population of fish, development gives rise to an ensemble of equivalent networks that differ in the precise number, morphology and connectivity of the constituent neurons, but whose overall function is strongly stereotyped. By smoothing away experimental variability, the low natural variation of low-dimensional dynamics within the wild type population enables us to observe the increased variation due to genetic alterations.

The δ2-pcdhs *pcdh19* and *pcdh17* are homophilic cell adhesion molecules that are linked to human neurodevelopmental disorders. Here we show that elimination of either *pcdh19* or *pcdh17* leads to altered population responses in the optic tectum. The neural response in the mutants deviates from wild type, with the specific differences varying among individual mutants. As a consequence, mutant individuals are much less similar to each other than are wild type larvae. This increased within-group variation implies that the loss of Pcdh19 or Pcdh17 does not lead to a particular, alternative network structure, where the variation would remain small, but to a stochastic array of alternatives. Reinforcing this idea, the deviations specific to each individual can be averaged away, as group averaged dynamics in the mutants closely matches the wild type averaged dynamics. Thus, the effects of the *pcdh19* and *pcdh17* mutations are best understood as an increase in stochastic developmental variation, leading to stochastic changes in network organization and increased phenotypic variability. The neural modes comprise the activity of individual orientation selective, direction selective and non-selective visually-responsive cells. The least affected of the neural modes is Mode 1, which appears to represent caudal-to-rostral motion, which is largely due to direction-selective retinal ganglion cell inputs [52,55], suggesting that the mutations primarily affect tectal neurons. Consistent with the changes in the neural modes, we show that the distributions of OSI and DSI among the mutants differ from wild type, as do the preferred directions of direction selective neurons. We attribute this to a broad degradation in the specificity of network connectivity. A recent study showed that morphological and functional properties vary within transcriptomically-defined neuronal types (t-types) [56] This suggests that even if the mutations affected a specific subset of t-types, the variation in functional properties within a t-type would lead to varying effects on the network. However, as *pcdh19* and *pcdh17* have broad expression within the tectum, it is likely that the mutations also impact a spectrum of t-types.

While both *pcdh19*^*-10 bp*^ and *pcdh19*^*Δprom*^ mutants result in a complete loss of Pcdh19 protein, their effects on the optic tectum are not identical. The phenotype associated with Pcdh19 loss in the *pcdh19*^*Δprom*^ line is significantly more severe than in the *pcdh19*^*-10 bp*^ line. Combined with the observation that the magnitude of the impact of indel mutations in *pcdh19*^*-10 bp*^ and *pcdh17*^*-5 bp*^ is similar, this suggests that the difference could be due to the activation of compensatory mechanisms by the indel mutations that are absent in the promoterless allele. This may indicate a further source of variability in understanding neural disorders and in the study of relevant genes in animal models, as even alternative “null” mutations may give rise to distinct phenotypes. Remarkably, mutants heterozygous for either of the *pcdh19* mutations exhibit phenotypes comparable to the corresponding homozygous mutants, indicating haploinsufficiency. Similar heterozygote phenotypes have been observed in zebrafish larvae lacking *pcdh19* [57] and for mice lacking *pcdh9* [58] or *pcdh10* [59]. The *pcdh17* heterozygotes are less affected than the *pcdh19* heterozygotes, but are still statistically different from wild type. Therefore, for δ-pcdhs, sibling heterozygotes should not be treated as controls, and single allele knock-in based reporters for these genes should not be considered wild type.

Visual responses in the optic tectum are first observed at 3 dpf [38], and larvae exhibit a variety of visually guided behaviors by 6 dpf [26,27,60]. During this time, neurogenesis and circuit assembly are ongoing [61]. Our results suggest that Pcdh19 and Pcdh17 contribute to developmental robustness of network assembly in the optic tectum [62]. As their loss leads to an increased variation in the neural response to visual stimulation, we suggest that this consequence could be considered an example of canalization [63,64]. Neural network assembly relies on stochastic neuronal dynamics in which axonal and dendritic branches extend to form synapses that may persist or be eliminated [37,65,66]. This iterative, exploratory process efficiently samples the pool of available synaptic partners, enabling the selection of correct synaptic connections [67–69]. Our data are consistent with the idea that the δ2-pcdhs bias this stochastic process. As δ2-pcdhs have been linked both to arbor growth and dynamics [70,71], and to synapse stability [72,73], they are ideally situated to influence network assembly. As we envision it, the impact of δ2-pcdh loss on neuronal tuning properties varies stochastically cell-by-cell, with the collective impact on the network varying among individual larvae, both in the particular effect and its severity.

A large proportion of neurodevelopmental disorders arise through the cumulative impact of common variants or pathogenic variants of small effect. The increased phenotypic variability observed in our mutants could represent the building block of this polygenicity. The stochastic variation that we see could be homologous to the variable penetrance and expressivity that characterizes weak risk alleles. The cumulative impact of multiple risk alleles could push individuals beyond a disease threshold in those instances where the superposition of phenotype happens to be larger. Thus, the approach that we present here could provide an effective means for investigating and quantifying polygenic effects.

## Methods

### Ethics statement

All animal procedures were performed in accordance with the Ohio State University Institutional Animal Care and Use Committee’s regulations (Protocol 2008A0226).

### Zebrafish maintenance and generation of lines

Adult zebrafish (Danio rerio) were maintained at ~28.5°C and staged according to Westerfield (1995). We previously generated lines harboring germline mutations of *pcdh19* (*pcdh19*^*os51*^) [25] and *pcdh17* (*pcdh17*^*os69*^) [49].

The *pcdh19*^*Δprom*^ mutant line (*pcdh19*^*os77*^) was established by co-injecting ribonucleoprotein complexes (Integrated DNA Technologies) consisting of Cas9 protein and gRNAs targeting a site in the 5’ end of *pcdh19* exon1 (GGGCTCAGATTAACCCATCG) and ~4 kb upstream of the ATG start codon (CTGTTGTGAGCTAGTTACCA), which is predicted to eliminate the promoter region. Founders were identified by PCR and sequenced, confirming the large genomic deletion. Like the *pcdh19*^*os51*^ line, Western blots confirmed that the homozygous *pcdh19*^*os77*^ line produced no Pcdh19 protein.

The plasmid *Tol2-elavl3:H2B-GCaMP8s* was assembled using the following plasmids: Tol2-elavl3-GCaMP6s (a gift from Misha Ahrens; Addgene plasmid # 59530; http://n2t.net/addgene:59530; RRID:Addgene_59530), CMV:Histone H2B-mGL (H2B-mGreenLantern was a gift from Gregory Petsko; Addgene plasmid # 164464; http://n2t.net/addgene:164464; RRID:Addgene_164464), and CMV:jGCaMP8s (gift from GENIE Project; Addgene plasmid # 162371; http://n2t.net/addgene:162371; RRID:Addgene_162371). Along with mRNA encoding Tol2 transpose, this plasmid was co-injected into 1 cell stage *nacre* embryos in order to establish the transgenic line *Tg(elavl3:H2B-GCaMP8s)*^*os78*^. This transgenic line was crossed with the *pcdh19*^*os51*^, *pcdh19*^*os77*^and *pcdh17*^*os69*^ mutant lines.

### HCR FISH staining

Two-color hairpin chain reaction fluorescent *in situ* hybridization (HCR FISH) was performed on 6 dpf *nacre* larvae. All the reagents including probes for *pcdh19*, *pcdh17*, *vglut2a* and *gad1b*, fluorescently labeled hairpins and buffers were purchased from Molecular Instruments (Los Angeles, CA, USA). The HCR FISH was performed according to Shainer et. al. 2023 [74]. For all experiments 10–12 embryos were incubated overnight at 37°C with probe solution containing 4 µl (1µM stock) of each of the two HCR probe sets in 500 µl of hybridization buffer. For the amplification step fluorescently labeled hairpin solution was prepared by combining snap-cooled 10 µl (3 µM stock) h1 and10 µl (3 µM stock) h2 hairpins for each of the probe sets in 500 µL of HCR amplification buffer. Embryos were incubated with hairpin solution overnight in the dark at room temperature. Excess hairpin solution was washed three times for 20 min each with 5X SSCT (5X sodium chloride sodium citrate + 0.1% tween20). After the final wash embryos were counterstained with DAPI (4,6-diamidino-2-phenylindole; Sigma-Aldrich, USA) at 200ng/ml and incubated overnight at 4°C with gentle shaking. Excess DAPI was washed with 5X SSCT and embryos were stored in the same solution at 4°C till ready to be imaged.

Embryos were embedded in 2% agarose in custom made glass bottom imaging chambers with #1.5 coverslip. Images were acquired using Zeiss LSM 900 Airyscan 2 equipped with water immersion objective (LD C-Apochromat 40X/ 1.1 NA). Image stacks were compiled using FIJI [75].

### Calcium imaging

Unanaesthetized 6 days-post-fertilization (dpf) larvae were embedded dorsal side up in 2% low melting point agarose. Imaging was performed on a custom-built resonant-scanning 2-photon microscope. Briefly, 920nm excitation was provided by an Axon920-TPC laser (Coherent, Inc.). We used a Nikon Apochromat 25x/1.1NA water-immersion objective for imaging. The resonant scan-head and controller, 3DMS robotic stage, Hamamatsu GaAsP photomultiplier tubes and power supply were obtained from Sutter Instruments (www.sutter.com). A piezo-electric objective positioner (nPFocus250) was obtained from nPoint (www.npoint.com). The microscope was run with ScanImage 5.2 [76] (Vidrio Technologies). All other parts were obtained from Thorlabs (www.thorlabs.com). Image stacks of 7 optical sections (512x512), spaced at 10 μm, were collected at 1 s intervals, with a pixel size of 0.65 μm. For each group, we included data from 9-13 larvae, which is in line with the number of fish used in comparable studies [39,77,78]. These were derived from at least two, separate crosses.

### Visual stimulation

Visual stimuli were programmed in Python, using PsychoPy3 (https://psychopy.org). Moving sinusoidal gratings were presented for 5 seconds, with 10 seconds of a neutral gray background in between each direction, which were rotated at 30° intervals. The stimulus set was presented three times, with 50 seconds in between each presentation. Stimuli were projected onto a translucent screen 2.5 cm from the larvae using a Rif6 cube picoprojector (rif6.com). The stimulus occupied ~104° of the visual field.

### Data analysis

We used CaImAn-MATLAB for processing of calcium imaging movies [41], including non-rigid motion correction with NoRMCorre [79]. First, sequences of image stacks (735 stacks of 7 planes) were re-formatted to movies of image planes (7 stacks of 735 timepoints). These were motion corrected and inspected for motion artifacts. Movies that exhibited z-drift or fish movements were discarded. Brightness and contrast were adjusted and then images were masked in order to analyze only the left (visually-responsive) hemi-tectum. CaImAn was used to segment movies and extract ΔF/F fluorescence traces. Cells that were responsive to the visual stimuli in each of the three trials were retained for further analysis.

Principal Component Analysis was performed on individual datasets and we projected the original neural responses onto these new basis vectors. As ~90% of the variance was explained by the top 5 principal components (neural modes), we restricted our analysis to these. While the first five neural modes were identified in each of our datasets, they were not always in the same order, as there was some fish-to-fish variation in how much variance was explained by each principal component. To facilitate comparisons, we placed the neural modes in a common order (S1 Fig), so that like activity patterns could be compared. In addition, the sign of a neural mode could vary among individual datasets, due to an arbitrary choice of direction for the principal component vectors during PCA; the neural data could be projected onto the positive or negative direction of the principal component. For comparisons of neural modes among datasets, we used Pearson’s correlation, r[47,48]. To compare trial-to-trial variability within larvae, the dynamics of each trial was correlated with each of the other trials, then these three comparisons were averaged (Figs 2D and S4). To compare between genotypes, the wild type dynamics along each mode were averaged to provide a common basis for comparison. Then each individual dataset was correlated with the wild type average (Figs 2F, 3C, 4B, and S8D). For the wild type dataset, we used a leave-one-out approach, where the data from each larva was compared to an average of the other eight larvae (Fig 2D). As an alternative measure of the variation in the datasets, each individual neural mode was correlated to all other equivalent modes within a group. This within-group pairwise correlation describes how much latent dynamics varies among individuals within a genotype (Figs 3D, 4C, and S8E). To provide a measure for the overall variance within a population, we pooled all pairwise correlation coefficients across all five modes and generated cumulative probability distributions (Fig 5A).

To determine the orientation and direction selectivity of neurons, we calculated an orientation selectivity index (OSI) and a direction selectivity index (DSI), with:


$$\begin{array}{c} \text{OSI }=\;({\text{F}}_{\text{preferred}}-{\text{F}}_{\text{orthogonal}})/({\text{F}}_{\text{preferred}}+{\text{F}}_{\text{orthogonal}}),\text{ and} \end{array}$$

$$\begin{array}{c} \text{DSI }=\;({\text{F}}_{\text{preferred}}-{\text{F}}_{\text{opposite}})/({\text{F}}_{\text{preferred}}+{\text{F}}_{\text{opposite}}), \end{array}$$

Where F_preferred_ is the maximal response to the 12 oriented stimuli and F_orthogonal_ and F_opposite_ are the magnitudes of the fluroscence signal at 90° and 180° from the direction of F_preferred_. To determine the preferred direction, we used the CircStat toolbox to calculate the mean direction from the circular data (https://github.com/circstat/circstat-matlab) [80]. Neurons were deemed direction selective, if DSI > 0.33. For the circular distributions, DSI values were placed in 30° centered around the 12 stimulus directions. The distributions are of cells pooled from all animals within a genotype.

For comparisons, statistical significance was determined using Dunnett’s test for multiple comparisons. For the comparison of distributions of r, we used a Dunn’s test. All statistical analysis was performed in JMP Pro 17 or JMP Student’s Edition 19, except circular distributions were compared with a MATLAB implementation of Watson’s U^2^ test (Pierre Mégevand (2025) pierremegevand/watsons_u2 https://github.com/pierremegevand/watsons_u2).

## Supporting information

S1 FigOrdering and alignment of dynamics.A,C Shown are reference dynamics along each mode (purple), and the dynamics in which neuronal responses from an individual larva were projected onto the first five neural modes obtained by PCA (teal). Apart from mode 1, the dynamics appear uncorrelated. However, the same patterns are present, but their relative order and sign differ. The order of modes varies due to variation in the relative importance of the principal components, and the sign varies, due to the chosen direction of the principal component when the neuronal responses are projected onto the axes. B. The dynamics can be sorted, so that each neural mode represents the same dynamics among the different fish (c). This includes adjusting the order of neural modes (arrows), as well as the sign (-1). Sorting is done by maximizing the correlation coefficients, r, for each neural mode. C Sorting the dynamics improves correlations, as only like neural modes are being averaged and compared.(TIF)

S2 Fig Generation of promoterless pcdh19 allele. A Schematic of the zebrafish *pcdh19* gene, with exons shown in purple. Pins show the sites of CRISPR/Cas9 target sites. Primers used for PCR-based screening are shown, as are the expected sizes of the PCR products. B Genotyping of mutant in-crosses using F1, F2 and R primers, which distinguish wild type, heterozygous and homozygous mutant larvae. C Western blot showing the absence of Pcdh19 protein in both our previously published mutant line and in the promoterless line presented here.
(TIF)

S3 Fig Expression of *pcdh19* and *pcdh17* in the optic tectum. A HCR-ISH showing the expression of *pcdh19* (green) and *vglut2a* (magenta), which is a marker of excitatory neurons (left panel, scale bar = 50 μm.). Pcdh19 is expressed in excitatory neurons both in neuropil interneurons (middle column, scale bar = 5 μm), and neurons in the stratum periventriculare (SPV) neurons (right column). DAPI is shown in blue. The overlap of green spots and blue shows up is displayed as cyan. White line highlights an individual cell. B HCR-ISH showing the expression of *pcdh19* (green) and *gad1b* (magenta), which is a marker of inhibitory neurons (left panel). Pcdh19 is expressed in inhibitory neurons both in neuropil interneurons (middle column), and neurons in the stratum periventriculare (SPV) neurons (right column). C HCR-ISH showing the expression of *pcdh17* (green) and *vglut2a* (magenta), which is a marker of excitatory neurons (left panel). Pcdh17 is expressed in excitatory neurons both in neuropil interneurons (middle column), and neurons in the stratum periventriculare (SPV) neurons (right column). D HCR-ISH showing the expression of *pcdh17* (green) and *gad1b* (magenta), which is a marker of inhibitory neurons (left panel). Pcdh17 is expressed in inhibitory neurons both in neuropil interneurons (middle column), and neurons in the stratum periventriculare (SPV) neurons (right column). E HCR-ISH showing the expression of *pcdh19* (green) and *pcdh17* (magenta) in the otpic tectum (left panel). Pcdh19 and Pcdh17 are expressed in distinct but overlapping sets of neurons. Examples of co-expression are shown both for neuropil interneurons (middle column), and neurons in the stratum periventriculare (SPV) neurons (right column).
(TIF)

S4 Fig Mutants do not exhibit increased trial-to-trial variability. Neural modes were determined from single trials and the average correlation across trials were determined for each larva. For *pcdh19*^Δprom^, Modes 1 and 3 showed a statistically significance difference in the trial-to-trial variability, but this did not correlate with the overall changes in the mutants.
(TIF)

S5 Fig Altered orientation selectivity in δ2-pcdh mutants. A-D. Cumulative probability distributions of orientation selectivity for all neurons in (A) wild type (purple; n = 9),(B) *pcdh19*^Δprom^ (teal; n = 9), (C) *pcdh19*^-10bp^ (blue; n = 12) and (D) *pcdh17*^-5bp^ (orange; n = 13) mutants. The distributions for individual fish are shown in thin lines and the distribution of the pooled data is shown as a thick line. As a reference, the pooled wild type data is shown in purple in each graph. Pcdh17 and Pcdh19 mutants exhibit distinct effects on orientation selectivity, with *pcdh17*^-5bp^ mutants exhibiting an overall shift toward reduced orientation selectivity, and both pcdh19 mutants exhibiting a right shift toward increased orientation selectivity.(**p < 0.0001; Dunn’s test).
(TIF)

S6 Fig Altered direction selectivity in δ2-pcdh mutants. A-D. Cumulative probability distributions of direction selectivity for all neurons in (A) wild type (purple; n = 9),(B) *pcdh19*^Δprom^ (teal; n = 9), (C) *pcdh19*^-10bp^ (blue; n = 12) and (D) *pcdh17*^-5bp^ (orange; n = 13) mutants. The distributions for individual fish are shown in thin lines and the distribution of the pooled data is shown as a thick line. As a reference, the pooled wild type data is shown in purple in each graph. Each of the mutants exhibit a decrease in direction selectivity, as the distributions show a left shift.(**p < 0.0001; Dunn’s test).
(TIF)

S7 Fig Distribution of preferred directions for direction selective neurons. A. A polar histogram showing the preferred directions of direction selective neurons (DSI > 0.33) in wild type larvae (n = 9). The preferred direction as obtained as the vector average of the responses across all stimuli. The distributions for individual larvae are shown in thin lines and the pooled data is shown by the thick line. B. A polar histogram showing the preferred direction of direction selective neurons in *pcdh19*^Δprom^ mutants (n = 9). The distributions for individual larvae are shown in thin lines and the pooled data is shown by the thick line. The distribution is significantly different from wild type (p < 0.0001; Watson’s U2 test). C. A polar histogram showing the preferred direction of direction selective neurons in *pcdh19*^-10bp^ mutants (n = 12). The distributions for individual larvae are shown in thin lines and the pooled data is shown by the thick line. The distribution is significantly different from wild type (p < 0.0001; Watson’s U2 test). D. A polar histogram showing the preferred direction of direction selective neurons in *pcdh17*^-5bp^ mutants (n = 13). The distributions for individual larvae are shown in thin lines and the pooled data is shown by the thick line. The distribution is significantly different from wild type (p < 0.0001; Watson’s U2 test).
(TIF)

S8 FigHeterozygous δ2-pcdh mutants exhibit altered neural dynamics.A-C. Latent dynamics computed from trial averaged neural data collected in heterozygous (A) *pcdh19*^Δprom^, (B) *pcdh19*^-10bp^ and (C) *pcdh17*^-5bp^ mutant larvae. Individual traces are shown on top of the group variance, shaded in blue. The value, $\overset{―}{\text{r}}$, represents the mean correlation of each individual neural mode to the wild type average of that neural mode. D. The correlation of individual trial-averaged neural modes to the wild type average (wild type, n = 9; *pcdh19*^Δprom^, n = 20; *pcdh19*^-10bp^, n = 14; *pcdh17*^-5bp^, n = 11). (*p < 0.05, Dunnett’s test). E. Pairwise within-group correlations of neural modes for wild type (purple) and *pcdh17* mutants (orange). (wild type, n = 36; *pcdh19*^Δprom^, n = 190; *pcdh19*^-10bp^, n = 91; *pcdh17*^-5bp^, n = 55; **p < 0.0001, *p,0.05, Dunnett’s test).(TIF)

S1 Video Calcium imaging of neural activity in response to visual stimulation. Visual stimulation was provided to the right eye (top) of a 6 dpf wild type larva. Fluorescence traces were extracted from cells in the left hemi-tectum (bottom half of optic tectum in video) for further analysis. Shown is a single plane from 7 planes collected at 1 second intervals for 735 second. Sequences of visual stimuli were repeated three times. The video has been motion corrected and adjusted for brightness and contrast.
(AVI)

S2 Video Calcium imaging of neural activity in a *pcdh19*^Δprom^ zebrafish larva. Shown is a single plane selected from an image stack of 7 planes collected at 1 second intervals for 735 second. Sequences of visual stimuli were repeated three times. The video has been motion corrected and adjusted for brightness and contrast.
(AVI)

S3 Video Calcium imaging of neural activity in a *pcdh19*^-10bp^ zebrafish larva. Shown is a single plane selected from an image stack of 7 planes collected at 1 second intervals for 735 second. Sequences of visual stimuli were repeated three times. The video has been motion corrected and adjusted for brightness and contrast.
(AVI)

S4 Video Calcium imaging of neural activity in *pcdh17*^-5bp^ zebrafish larva. Shown is a single plane selected from an image stack of 7 planes collected at 1 second intervals for 735 second. Sequences of visual stimuli were repeated three times. The video has been motion corrected and adjusted for brightness and contrast.
(AVI)

S1 Data Includes the data values used in Fig 2 and 2F. (XLSX)

S2 Data Includes the data and p-values used in Fig 3 and 3D. (XLSX)

S3 Data Includes the data and p-values used in Fig 4 and 4C. (XLSX)

S4 Data Includes the data and p-values used in Fig 5A. (XLSX)

S5 Data Includes the data and p- values used in S4 Fig. (XLSX)

S6 Data Includes the data p-values used in S5 Fig. (XLSX)

S7 Data Includes the data p-values used in S6 Fig. (XLSX)

S8 Data Includes the data p-values used in S7 Fig. (XLSX)

S9 Data Includes the data p-values used in S8 and S8E Fig. (XLSX)
