# Supplementary figures and images for: Canalization of neural dynamics by δ-protocadherins in the developing zebrafish optic tectum
Source: PLoS Genet. 2026 Jun 1;22(6):e1012171. doi: 10.1371/journal.pgen.1012171 (PMC13241009; doi:10.1371/journal.pgen.1012171)

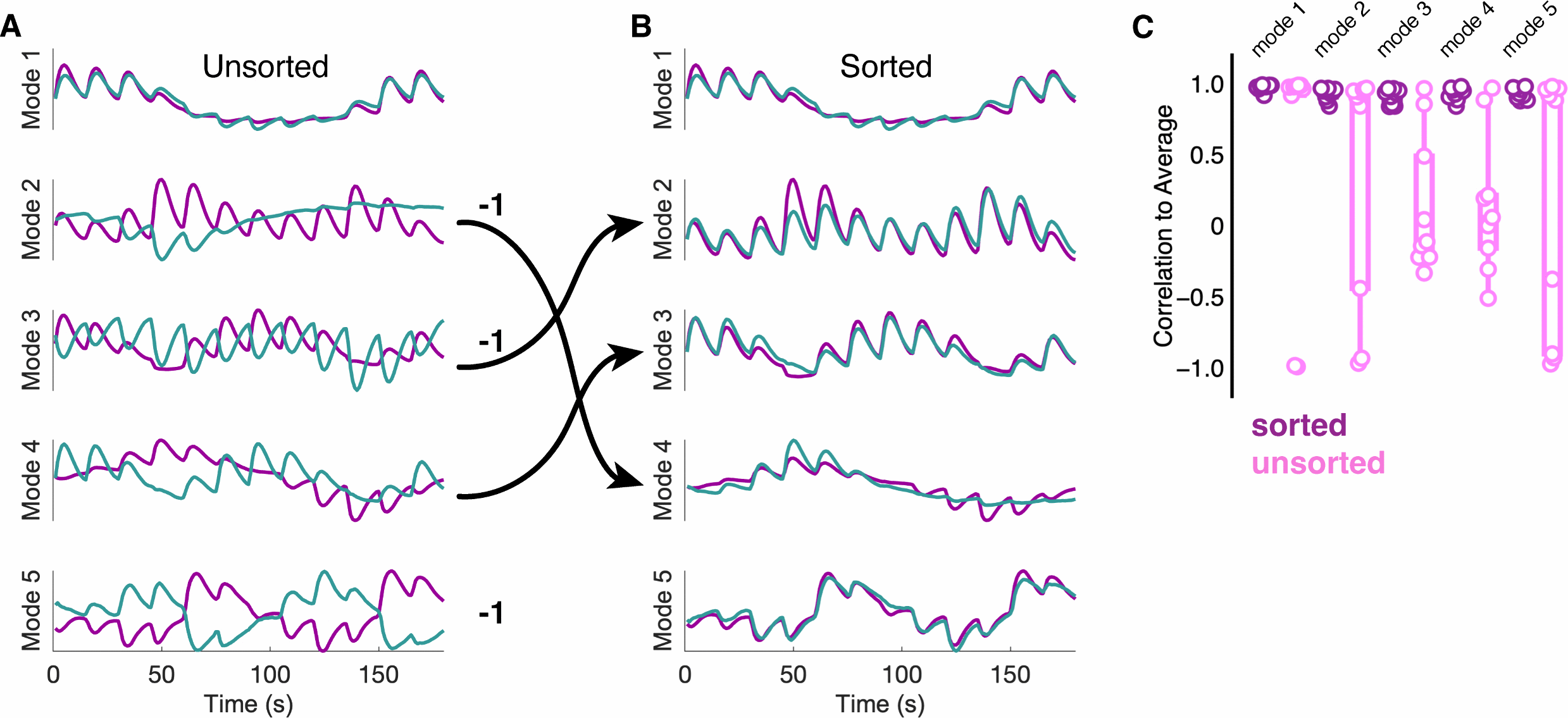

Supplement: S1 Fig — A,C Shown are reference dynamics along each mode (purple), and the dynamics in which neuronal responses from an individual larva were projected onto the first five neural modes obtained by PCA (teal). Apart from mode 1, the dynamics appear uncorrelated. However, the same patterns are present, but their relative order and sign differ. The order of modes varies due to variation in the relative importance of the principal components, and the sign varies, due to the chosen direction of the principal component when the neuronal responses are projected onto the axes. B. The dynamics can be sorted, so that each neural mode represents the same dynamics among the different fish (c). This includes adjusting the order of neural modes (arrows), as well as the sign (-1). Sorting is done by maximizing the correlation coefficients, r, for each neural mode. C Sorting the dynamics improves correlations, as only like neural modes are being averaged and compared. (TIF) [file pgen.1012171.s001.tif]

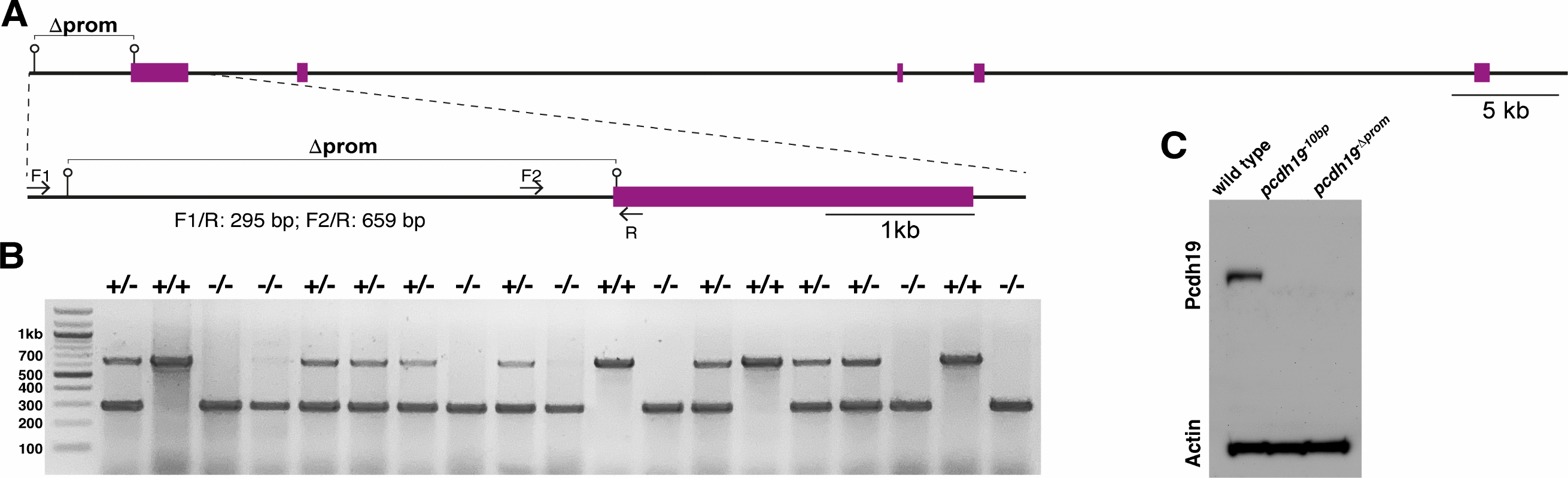

Supplement: S2 Fig — A Schematic of the zebrafish pcdh19 gene, with exons shown in purple. Pins show the sites of CRISPR/Cas9 target sites. Primers used for PCR-based screening are shown, as are the expected sizes of the PCR products. B Genotyping of mutant in-crosses using F1, F2 and R primers, which distinguish wild type, heterozygous and homozygous mutant larvae. C Western blot showing the absence of Pcdh19 protein in both our previously published mutant line and in the promoterless line presented here. (TIF) [file pgen.1012171.s002.tif]

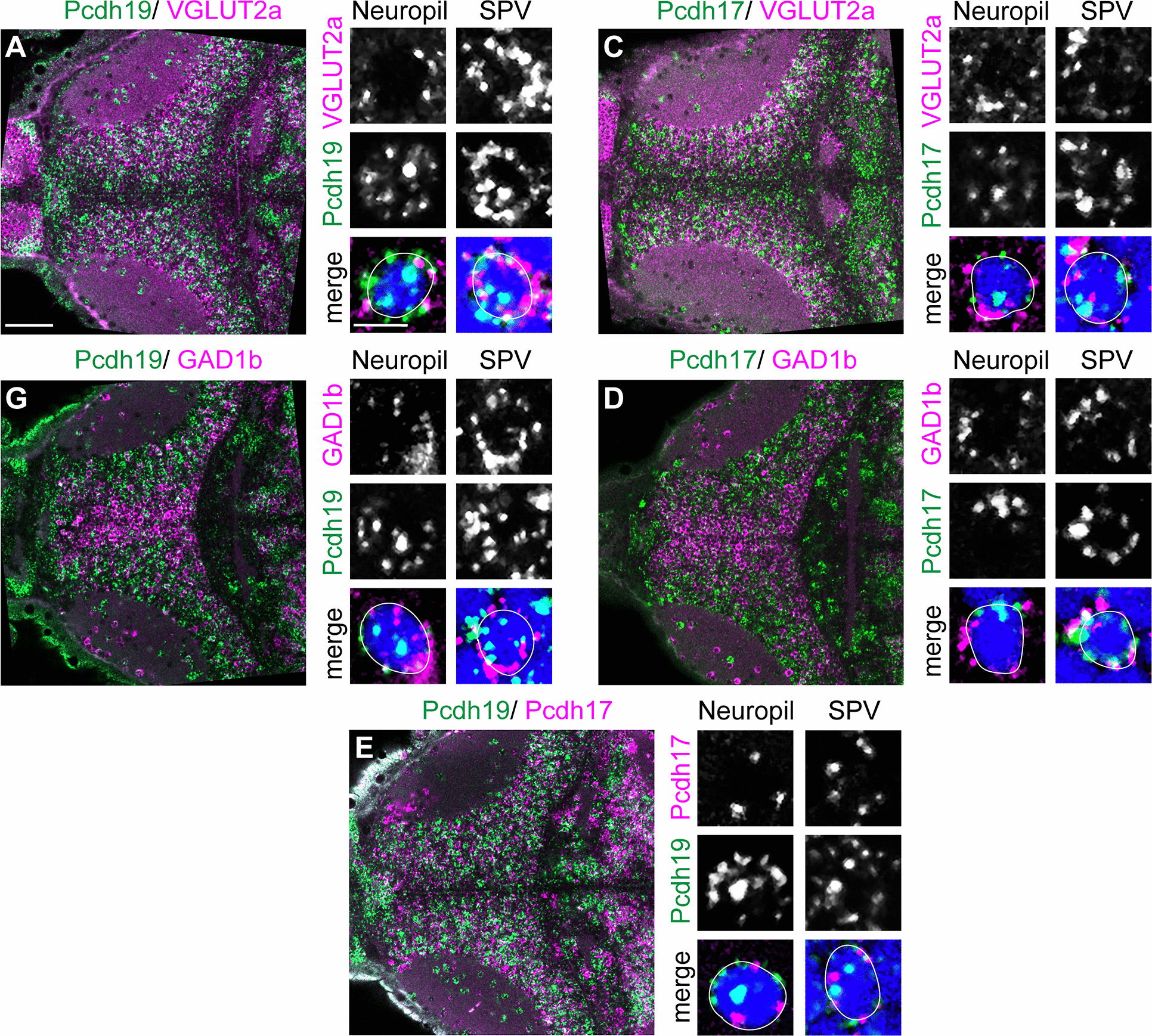

Supplement: S3 Fig — A HCR-ISH showing the expression of pcdh19 (green) and vglut2a (magenta), which is a marker of excitatory neurons (left panel, scale bar = 50 μm.). Pcdh19 is expressed in excitatory neurons both in neuropil interneurons (middle column, scale bar = 5 μm), and neurons in the stratum periventriculare (SPV) neurons (right column). DAPI is shown in blue. The overlap of green spots and blue shows up is displayed as cyan. White line highlights an individual cell. B HCR-ISH showing the expression of pcdh19 (green) and gad1b (magenta), which is a marker of inhibitory neurons (left panel). Pcdh19 is expressed in inhibitory neurons both in neuropil interneurons (middle column), and neurons in the stratum periventriculare (SPV) neurons (right column). C HCR-ISH showing the expression of pcdh17 (green) and vglut2a (magenta), which is a marker of excitatory neurons (left panel). Pcdh17 is expressed in excitatory neurons both in neuropil interneurons (middle column), and neurons in the stratum periventriculare (SPV) neurons (right column). D HCR-ISH showing the expression of pcdh17 (green) and gad1b (magenta), which is a marker of inhibitory neurons (left panel). Pcdh17 is expressed in inhibitory neurons both in neuropil interneurons (middle column), and neurons in the stratum periventriculare (SPV) neurons (right column). E HCR-ISH showing the expression of pcdh19 (green) and pcdh17 (magenta) in the otpic tectum (left panel). Pcdh19 and Pcdh17 are expressed in distinct but overlapping sets of neurons. Examples of co-expression are shown both for neuropil interneurons (middle column), and neurons in the stratum periventriculare (SPV) neurons (right column). (TIF) [file pgen.1012171.s003.tif]

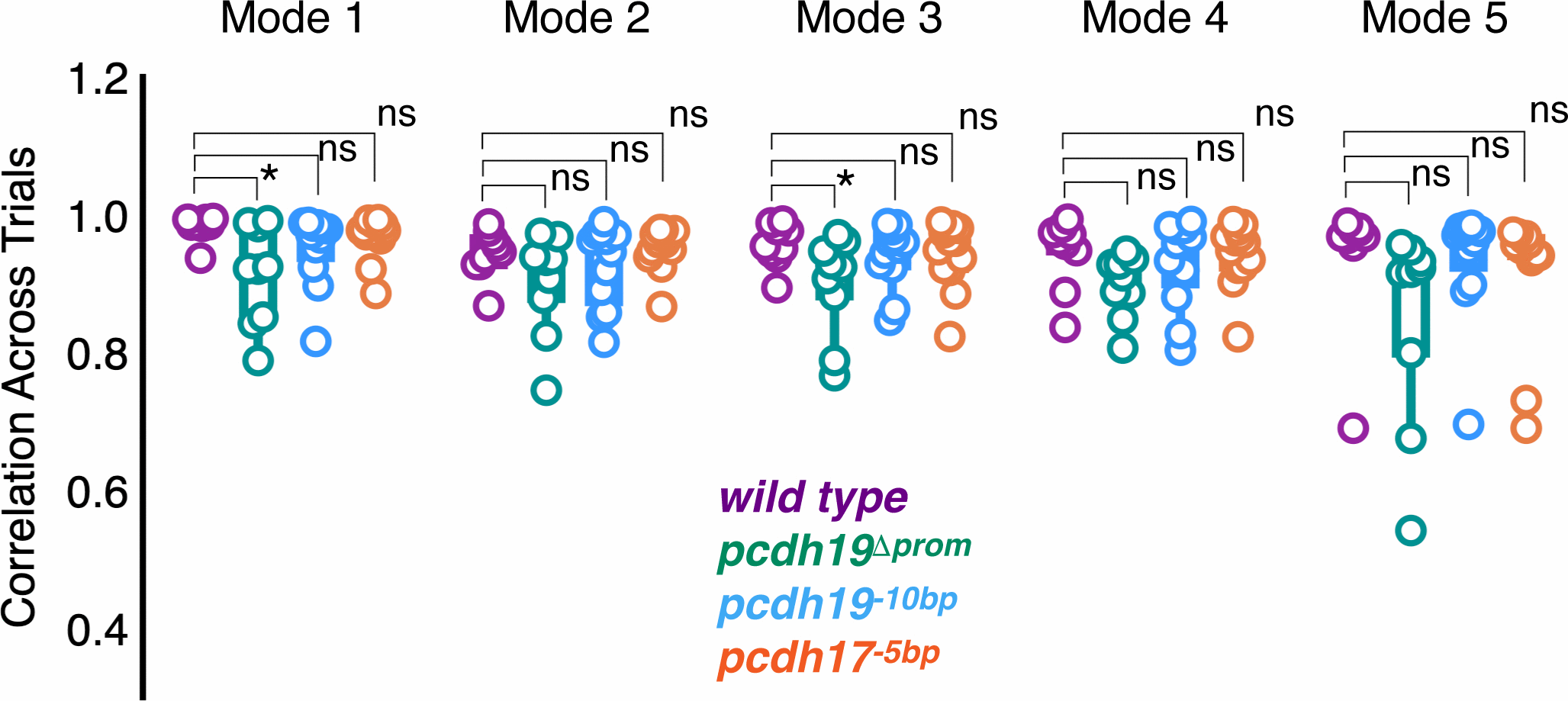

Supplement: S4 Fig — Neural modes were determined from single trials and the average correlation across trials were determined for each larva. For pcdh19Δprom, Modes 1 and 3 showed a statistically significance difference in the trial-to-trial variability, but this did not correlate with the overall changes in the mutants. (TIF) [file pgen.1012171.s004.tif]

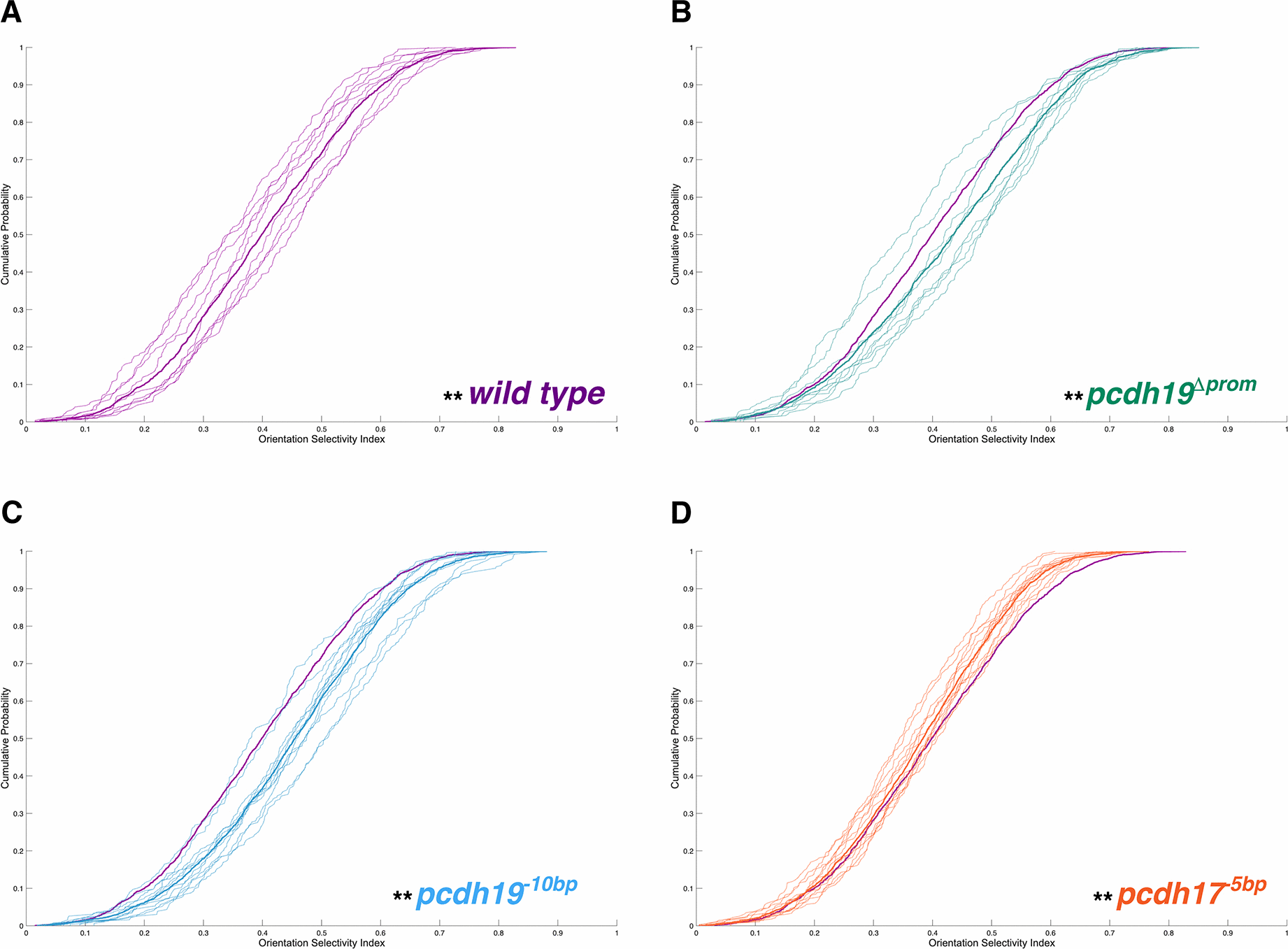

Supplement: S5 Fig — A-D. Cumulative probability distributions of orientation selectivity for all neurons in (A) wild type (purple; n = 9),(B) pcdh19Δprom (teal; n = 9), (C) pcdh19-10bp (blue; n = 12) and (D) pcdh17-5bp (orange; n = 13) mutants. The distributions for individual fish are shown in thin lines and the distribution of the pooled data is shown as a thick line. As a reference, the pooled wild type data is shown in purple in each graph. Pcdh17 and Pcdh19 mutants exhibit distinct effects on orientation selectivity, with pcdh17-5bp mutants exhibiting an overall shift toward reduced orientation selectivity, and both pcdh19 mutants exhibiting a right shift toward increased orientation selectivity.(**p < 0.0001; Dunn’s test). (TIF) [file pgen.1012171.s005.tif]

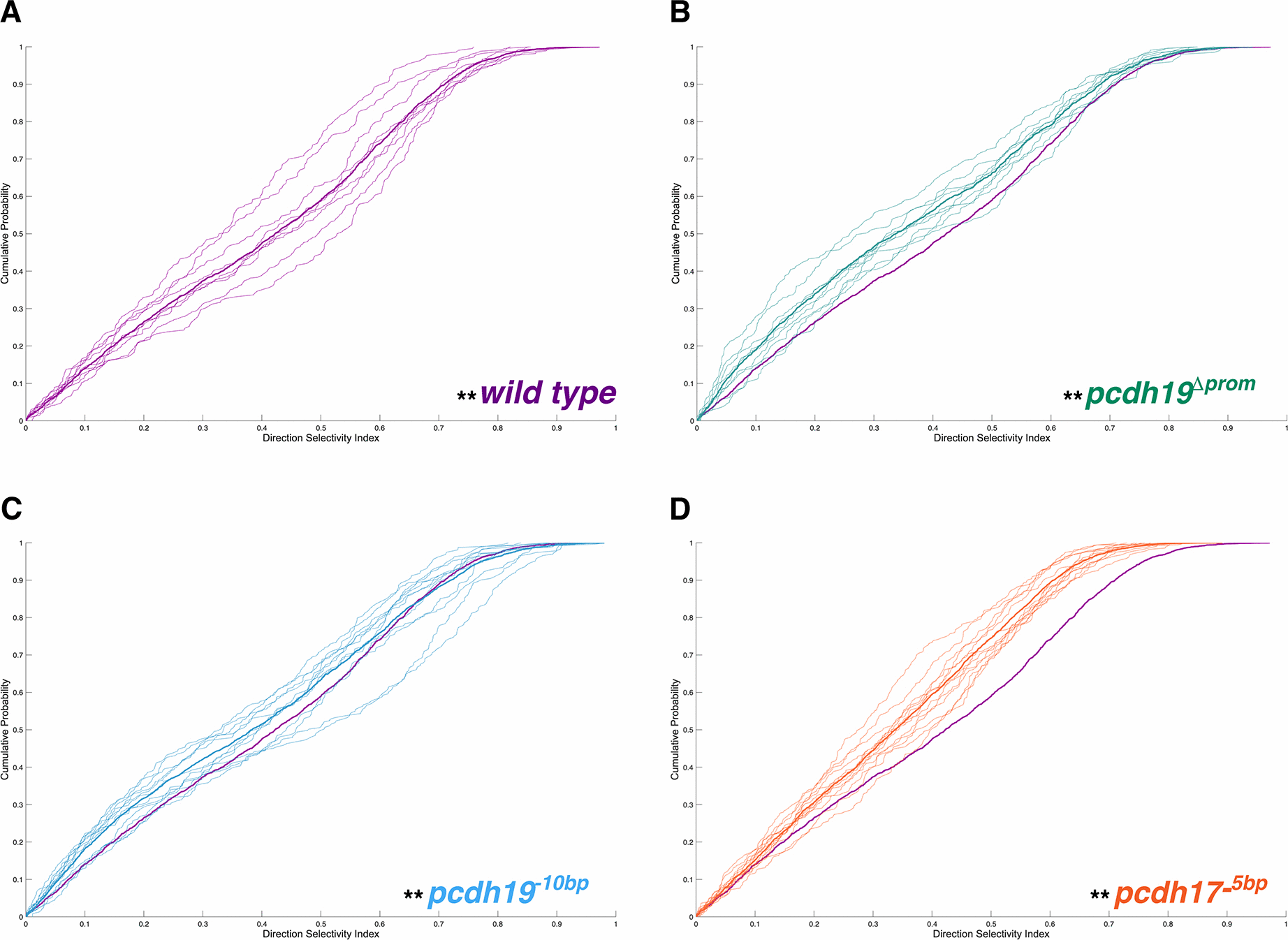

Supplement: S6 Fig — A-D. Cumulative probability distributions of direction selectivity for all neurons in (A) wild type (purple; n = 9),(B) pcdh19Δprom (teal; n = 9), (C) pcdh19-10bp (blue; n = 12) and (D) pcdh17-5bp (orange; n = 13) mutants. The distributions for individual fish are shown in thin lines and the distribution of the pooled data is shown as a thick line. As a reference, the pooled wild type data is shown in purple in each graph. Each of the mutants exhibit a decrease in direction selectivity, as the distributions show a left shift.(**p < 0.0001; Dunn’s test). (TIF) [file pgen.1012171.s006.tif]

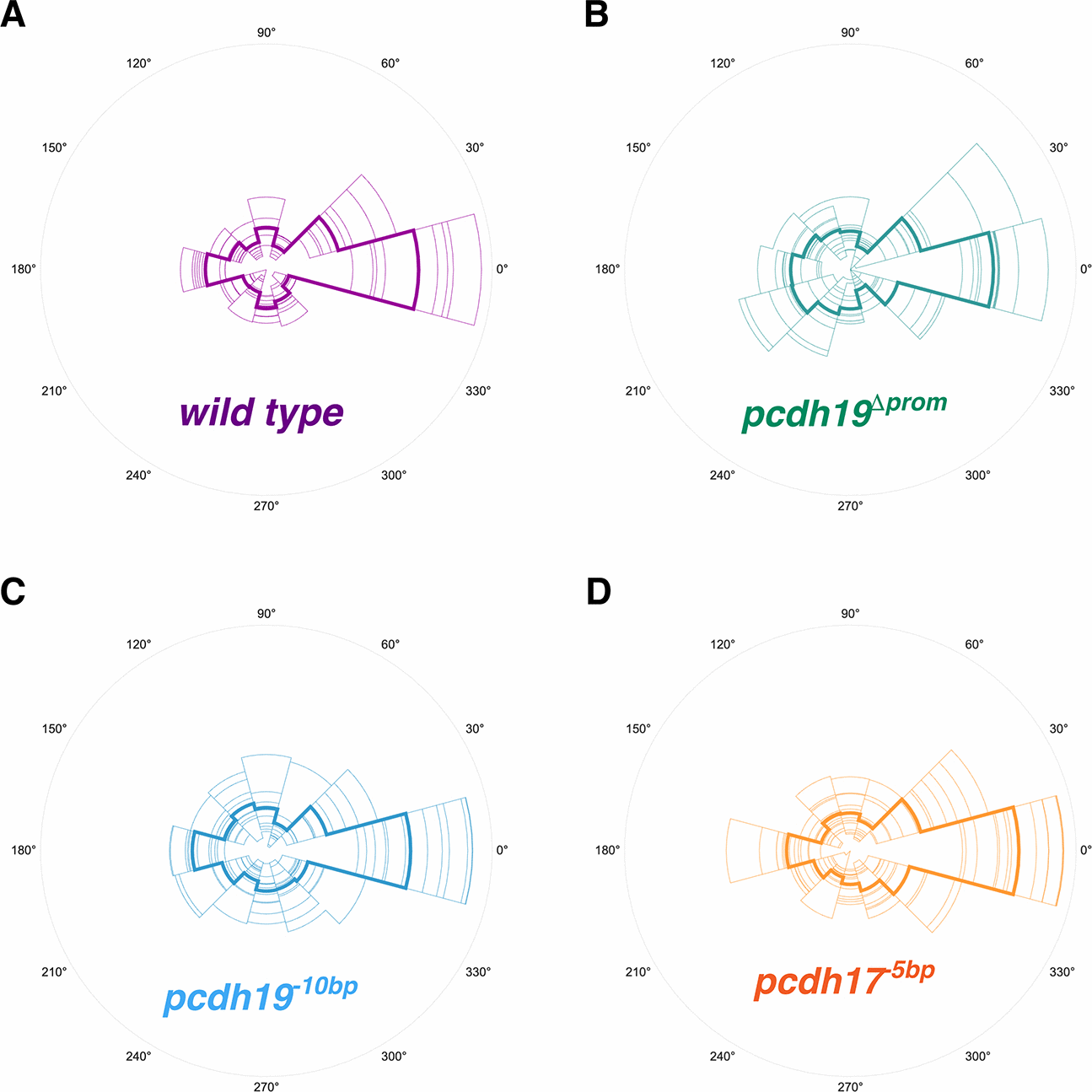

Supplement: S7 Fig — A. A polar histogram showing the preferred directions of direction selective neurons (DSI > 0.33) in wild type larvae (n = 9). The preferred direction as obtained as the vector average of the responses across all stimuli. The distributions for individual larvae are shown in thin lines and the pooled data is shown by the thick line. B. A polar histogram showing the preferred direction of direction selective neurons in pcdh19Δprom mutants (n = 9). The distributions for individual larvae are shown in thin lines and the pooled data is shown by the thick line. The distribution is significantly different from wild type (p < 0.0001; Watson’s U2 test). C. A polar histogram showing the preferred direction of direction selective neurons in pcdh19-10bp mutants (n = 12). The distributions for individual larvae are shown in thin lines and the pooled data is shown by the thick line. The distribution is significantly different from wild type (p < 0.0001; Watson’s U2 test). D. A polar histogram showing the preferred direction of direction selective neurons in pcdh17-5bp mutants (n = 13). The distributions for individual larvae are shown in thin lines and the pooled data is shown by the thick line. The distribution is significantly different from wild type (p < 0.0001; Watson’s U2 test). (TIF) [file pgen.1012171.s007.tif]

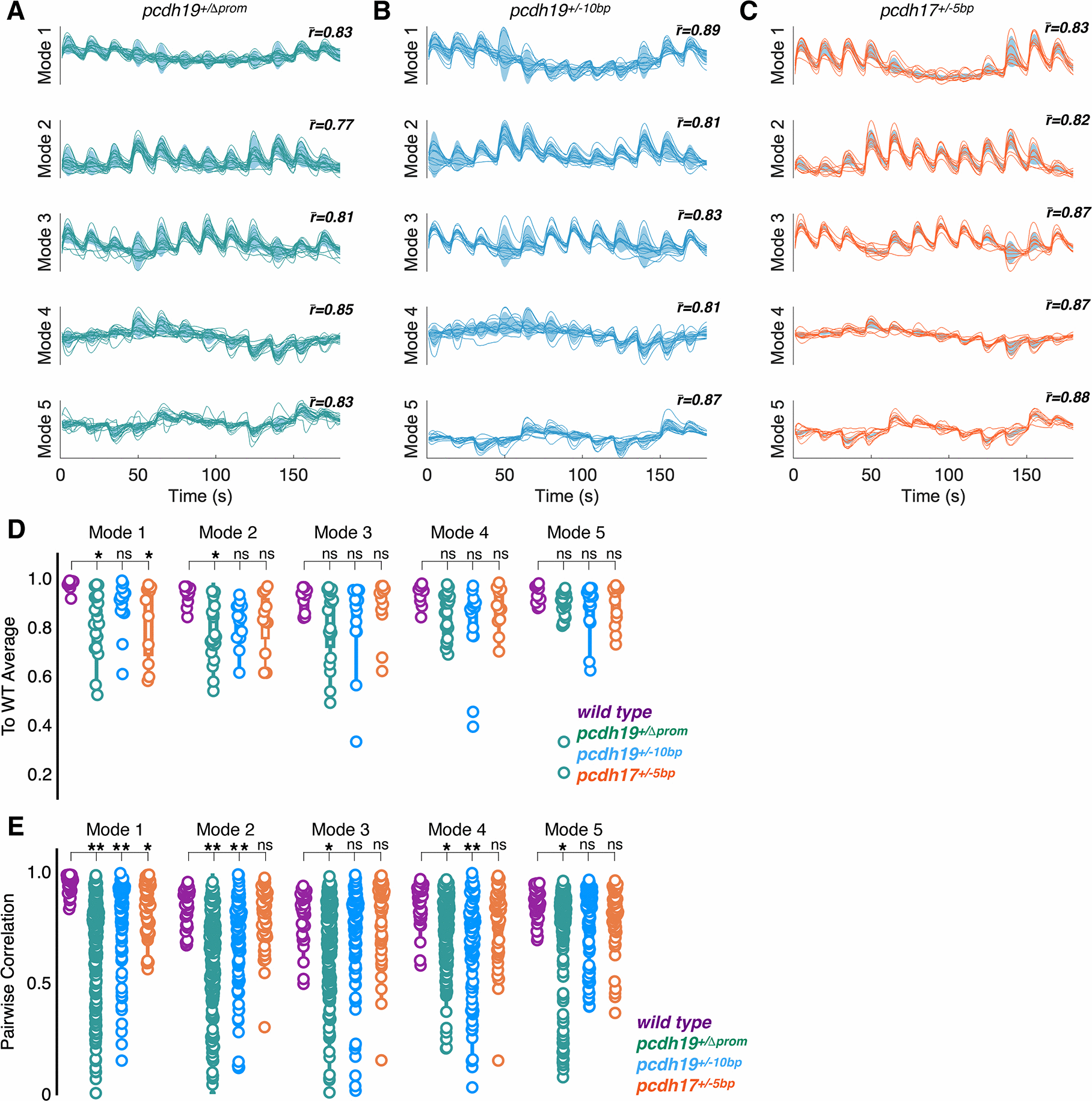

Supplement: S8 Fig — A-C. Latent dynamics computed from trial averaged neural data collected in heterozygous (A) pcdh19Δprom, (B) pcdh19-10bp and (C) pcdh17-5bp mutant larvae. Individual traces are shown on top of the group variance, shaded in blue. The value, r―, represents the mean correlation of each individual neural mode to the wild type average of that neural mode. D. The correlation of individual trial-averaged neural modes to the wild type average (wild type, n = 9; pcdh19Δprom, n = 20; pcdh19-10bp, n = 14; pcdh17-5bp, n = 11). (*p < 0.05, Dunnett’s test). E. Pairwise within-group correlations of neural modes for wild type (purple) and pcdh17 mutants (orange). (wild type, n = 36; pcdh19Δprom, n = 190; pcdh19-10bp, n = 91; pcdh17-5bp, n = 55; **p < 0.0001, *p,0.05, Dunnett’s test). (TIF) [file pgen.1012171.s008.tif]
